# Performance of Building Solid Waste Powder in Cement Cementitious Material: A Review

**DOI:** 10.3390/ma15155408

**Published:** 2022-08-05

**Authors:** Yongcheng Ji, Wenhao Ji, Wei Li

**Affiliations:** School of Civil Engineering, Northeast Forestry University, Harbin 150040, China

**Keywords:** recycled powder (RP), cement cementitious material, mechanical properties, early performance, regression analysis

## Abstract

Recycled powder (RP) is a by-product of preparing recycled aggregates from construction waste through debris removal, step-by-step crushing, screening, and mechanical strengthening. It is a fine powder with a particle size of less than 75 μm. Reasonable use of RP can increase the utilization rate of construction waste and reduce dust pollution. This study introduces the current research status of RP. It describes the source of RP; the activation mode of activity; the effect on several aspects, such as early performance and mechanical properties of cement-based materials; and its mechanism of action in light of the research and development. Moreover, the linear regression analysis method was used to obtain the mathematical model between the content of RP and the performance of cement-based materials. The correlation degree between the content of RP and the performance of cement-based materials was obtained based on the gray relation analysis method. It was concluded that the change of the content of RP had the most significant influence on the compressive strength of foamed concrete over 28 d. Finally, some feasible suggestions and prospects for RP are provided.

## 1. Introduction

With the rapid development of the economy, the ecological environment, natural resources, and other problems have become increasingly prominent. According to the estimation of relevant industry associations, China’s annual waste production has exceeded 2 billion tons in recent years. As a result, China’s annual production scale of waste reached 2.8 billion tons in 2020. It is expected to reach 7.3 billion tons in 2030, with construction waste already accounting for 40% to 50% of total waste [1]. Construction waste is usually disposed of by landfill or incineration, which inevitably causes many environmental problems, such as air quality and soil pollution.

In the 1980s, Buck pointed out the use of concrete waste from construction waste to prepare recycled aggregates for the green recycling of construction waste. This idea was quickly recognized, and research on the green recycling of construction waste continues today [2]. However, some developed countries have done a better job in this area, and they have reached 60% to 97% waste resource utilization rate. Among them, the European Union (90%), Japan (97%), and Korea (97%) have reached a reasonably high level [3]. For example, South Korea’s “Lifom System” decoration company separates the cement, sand, and steel in the concrete, then heats them at a high temperature of 700 °C and adds particular substances to produce recycled cement. This recycled cement complies with Korean construction standards and produces no carbon dioxide during production [4].

Currently, there are two main methods of resource utilization of construction waste: (1) preparation of recycled concrete, recycled bricks, and other construction materials. For example, Wu [5] et al. used construction brick mix waste to prepare recycled concrete pavement blocks and tested their basic mechanical properties early. The research results showed that the recycled concrete pavement bricks prepared using the raw materials and mixing ratio of construction brick and concrete waste met the requirements of relevant specifications. Soni [6] et al. used construction waste to produce crushed recycled coarse aggregate (CRCA) to replace natural sand as fine aggregates. The study demonstrated that concrete with improved compressive strength could be produced with 100% incorporation of specific CRCA as fine aggregate. (2) Used in road base pavement, foundation treatment, or subsidiary settings in road projects. For example, Cosenza [7] et al. applied recycled aggregates prepared from construction waste (mainly basalt, sandstone, and structural concrete) instead of natural aggregates in road subgrade. The research showed that the recycled mixes had good mechanical properties for road subgrade construction. Li [8] studied the road performance of construction waste fine aggregates applied to road base backfill materials. The results showed that both recycled aggregates of concrete block and brick structure could meet the strength requirements of road base materials when the compaction meets the specification requirements.

In the process of recycling construction waste, many scholars prepare it into recycled aggregates to be utilized. However, a certain amount of fine powder with a particle size of less than 75 μm, called recycled powder (RP), will be produced with this preparation of recycled aggregates. The use of RP is of great significance for the full recycling of construction waste. Schoon et al. [9] and Park et al. [10], found that RP contains some active substances and a large amount of SiO_2_, and the results of the study showed that 10% to 15% of the cement can be replaced by the same mass of RP in concrete. However, this untreated RP will reduce the performance of the concrete. Cwirzen [11] and Han-Young et al. [12] found that the addition of RP reduced the mechanical properties of concrete, and Singh [13] et al. also established a mathematical model of marble powder admixture and 28 d compressive strength of concrete. Foreign scholars also studied the influence of RP incorporation on concrete durability in detail. Kim [14] and Sabina [15] et al. found that the carbonation depth was positively correlated with the amount of RP in their experimental studies. Ann [16] tested that exposing recycled brick powder (RBP) concrete to high temperatures can improve the strength of concrete, and Lam [17] reported that adding 20–45% recycled brick powder (RBP) can improve the heat resistance of concrete.

Compared with developed countries, China started relatively late in this field, but domestic scholars have also made specific achievements. Mao [18] studied the chemical composition of recycled powder, fly ash, and cement. The three substances were similar in composition, differing only in the contents of Al_2_O_3_, CaO, and SiO_2_. It was inferred that RP has class activity and is feasible as a cementitious material instead of cement. Yu [19] compared two different mechanical crushing methods—airflow crushing and vibratory ball mill crushing—to deal with RP. It was found that the airflow-crushed RP had more uniform particles and higher activity. The mortar’s compressive strength was higher than the RP mixed with vibratory ball mill grinding. RP’s admixture was increased to improve its utilization rate and utilization range. Zhang [20] found that when the fine recycled powder content is less than 20%, the higher the content, the better the frost resistance. For resistance to sulfate erosion, the higher the content of RP, the more serious the sulfate erosion.

This paper expounds on RP’s research status and applications, introduces RP’s influence and mechanism on the early performance and mechanical properties of cement-based materials in detail, and summarizes RP’s source, preparation, and activation methods. Furthermore, the linear regression analysis method was used to obtain the mathematical model between the content of RP and the performance of cement-based materials. Furthermore, the correlation degree between the content of RP and the performance of cement-based materials was obtained based on the gray relation analysis method. Finally, some thoughts and ideas are presented.

## 2. Source and Preparation of RP

### 2.1. Source of RP

According to the difference in the scale of preparation, the sources of RP can be divided into two categories: (1) small-scale preparation in the laboratory using waste cement-based materials to grind and prepare RP. The advantage of this RP is that the mixing ratio, age, and other factors of the raw materials can be clearly understood, which can provide reliable support for the later analysis of the experimental results. However, its disadvantages are also very obvious. Due to the varying level of laboratory equipment, the particle size distribution and fineness of the RP produced cannot be guaranteed, and the performance varies significantly. (2) Large-scale standardized preparation in factories. The advantage of this RP is that various parameters such as fineness and particle shape can be controlled through a standardized preparation process. Its disadvantage is that the composition of construction waste is complex. As a result, the performance parameters of the original material of the RP cannot be traced. 

According to the different original materials, the sources of RP can be divided into three categories: (1) the fine powder particles obtained by the crushing, screening, and grinding of waste concrete are called recycled concrete powder (RCP). (2) Discarded bricks or red bricks after debris removal crushed and ground to achieve a particle size of less than 75 μm, known as a recycled brick powder (RBP). (3) The fine powder produced from the waste cement mortar after a series of processes such as crushing and grinding is called recycled mortar powder (RMP). These three different powders together make up the RP.

### 2.2. Preparation of RP

Regarding the preparation of RP, extensive research has also been conducted by domestic and foreign scholars. Li et al. [21] made ultrafine powder with coal gasification fine slag from seven regions as raw material. The influence of ball milling time was investigated when subjected to the particle size, specific surface area, microstructure, and phase composition of ultrafine powder. Results showed that the particle size of ultrafine powder changed before ball milling, and the specific surface area increased to 890 m^2^/kg when particle size decreased. With the increase of ball milling time, more spherical particles change into irregular, and angular particles, the crystal phase strength of ultrafine powder decreases the diffraction peak width increases, and it has higher reactivity. Liu [22] used aerated concrete blocks and sintered clay bricks to prepare RP. The study showed that aerated concrete blocks were more accessible to break than sintered clay bricks. However, the energy consumption of grinding aerated concrete blocks to less than 0.30 mm powder was higher than that of sintered clay brick powder. In their experiment, Liu [23], Huang [24], et al. used the RP generated in the production of recycled aggregate by construction waste in the factory. However, this kind of regenerated micro powder has a small specific surface area and low activity, which often needs to be activated in the later use process. Fu [25], Kang [26], Zhao [27], et al. used RP from discarded beams and concrete column components in their experiment in the laboratory after step-by-step crushing, screening, and grinding.

**Figure 1:**
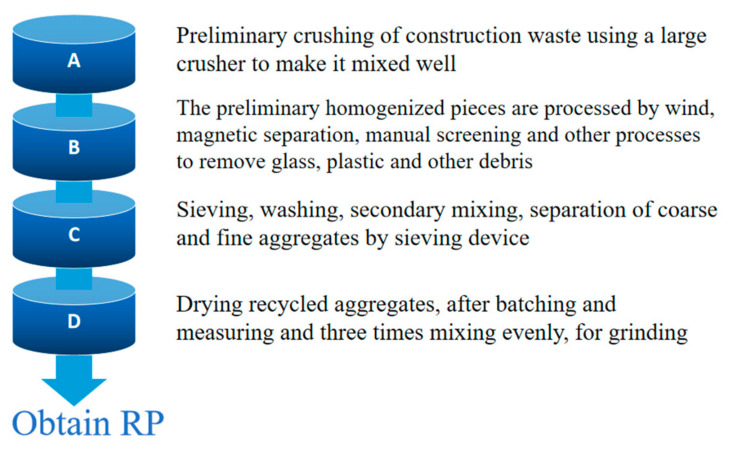
Process of industrial preparation of RP.

**Figure 2:**
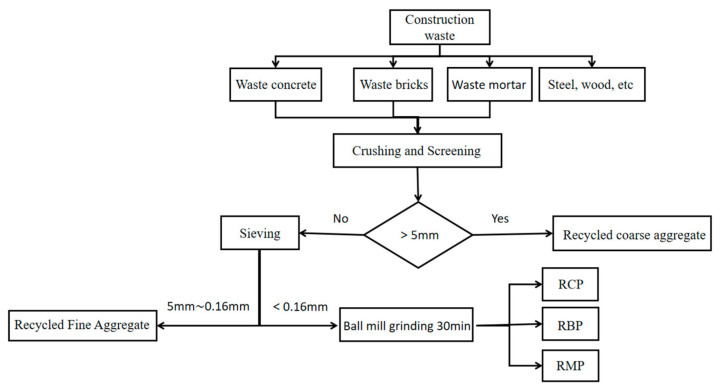
Preparation process of RP in laboratory.

**Figure 3:**
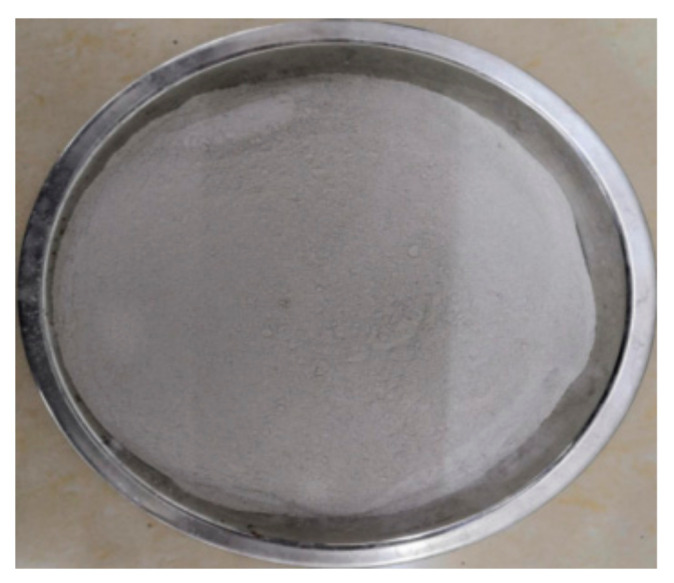
RCP [29].

**Figure 4:**
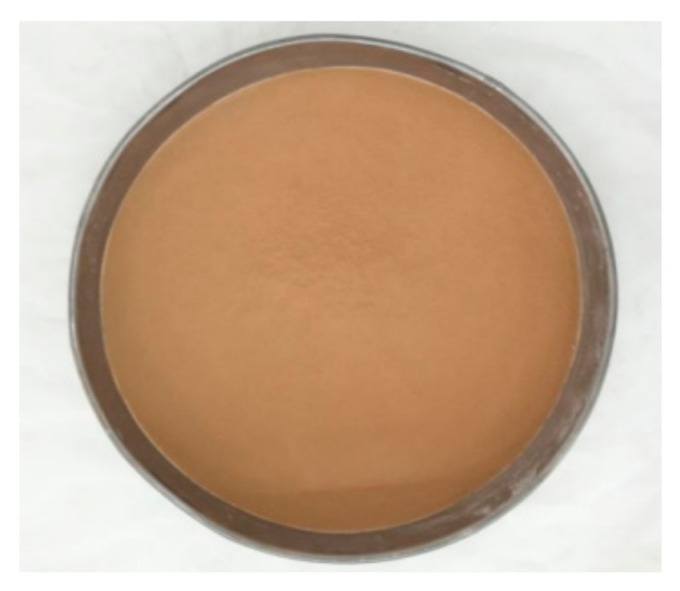
RBP [30].

**Figure 5:**
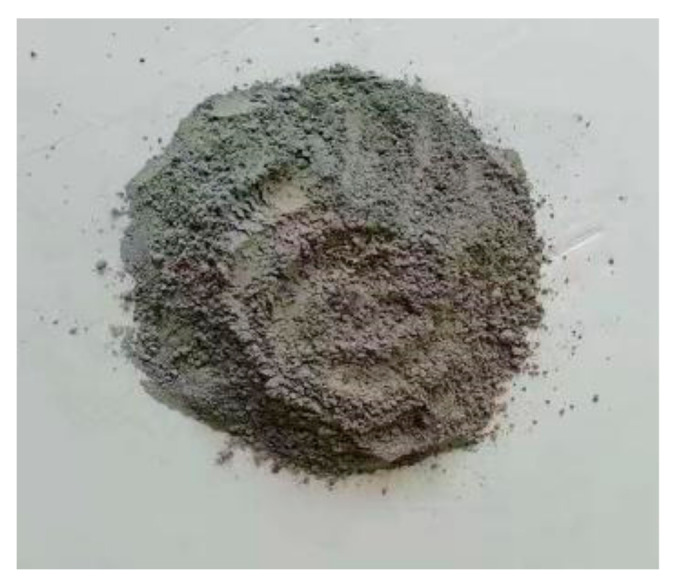
RMP.

The preparation process of RP can be divided into three types according to different equipment conditions and preparation methods: (1) The RP was obtained by crushing, sieving, grinding, and processing and preparing from waste concrete in the laboratory. (2) Prepared by specialized building materials enterprises using standard industrial processes. The larger volume of construction waste is crushed for the first time, followed by removing impurities such as plastic, steel, and woodblocks from the waste using wind and magnetic separation. The impurities in the construction waste are first screened, then sorted for the second time by manual screening. Then, the mud and mortar on the surface of the fragments are separated by a professional grinding machine. The screening system crushed the coarse aggregates of different particle sizes for the second time; in the fourth step, the aggregates were ground into powder by the grinding equipment [28]. The process of industrial preparation of RP is shown in Figure 1 below. (3) RP is obtained by collecting byproducts from the preparation of regenerated aggregate. Figure 2 below shows the flow chart of the preparation of RP. Figure 3, Figure 4 and Figure 5 show the prepared RCP, RBP, and RMP, respectively.

In RP preparation, the grinding stage is crucial, and the grinding equipment and grinding time play an essential role in the performance of RP. Fediuk et al. [31] invented a grinding machine that can grind powders to nano-scale, resulting in more uniform particles and better gradation than ordinary grinding equipment. Yu [19] ground the same RP using an ultra-micro airflow crusher for 30 min and vibrating ball mill for 120 min. The RP treated with the airflow crusher was active, and it was added to the mortar instead of being used as part of the cement to enhance the hydration of the cementitious material. This can promote the strength of the mortar. The grinding time also affects the performance of the RP. Different grinding equipment grinds for different times, and the performance of the RP obtained varies. The length of grinding time determines the RP’s fineness and specific surface area. Wang et al. [32] used a ball mill to grind waste concrete particles for 60, 90, and 120 min. The specific surface areas of the powder particles were 389.32 m^2^/kg^−1^, 464.51 m^2^/kg^−1^, and 467.08 m^2^/kg^−1^, respectively. Yu et al. [33] used a crusher and a conical ball mill to make RP from brick and concrete construction waste. Its average diameter was controlled below 40 μm, which improved recycled fine powder use efficiency in concrete. Li et al. [34] found that using a ball mill to grind RP, the amount of RP with a particle size of less than 20 μm increased continuously with the increase in grinding time. When the grinding time exceeded 20 min, the grinding efficiency decreased, and the RP obtained at 30 min had the highest activity. The grinding time is not as long as possible, and beyond a certain time, continued grinding will only result in a waste of resources and has little effect in improving the activity of the RP. The optimal grinding time is related to the type of grinding equipment, the type of RP, the initial particle size of RP, and other factors. The current research on this aspect is still slightly insufficient. Determining the optimal grinding time for different equipment and different types of RP is of great significance for the industrial production of RP.

## 3. Fundamental Properties of RP

### 3.1. Physical Properties of RP

The physical properties of recycled RP vary somewhat depending on the preparation method and the source of the material. The apparent density of RP is generally 2300 kg/m^3^ to 2700 kg/m^3^, the specific surface area is 300 m^2^/kg to 700 m^2^/kg, and the bulk density is 870 kg/m^3^ to 920 kg/m^3^ [35,36,37,38,39]. Regarding the particle size of RP, due to differences in experimental materials and instruments, domestic scholars have reached different conclusions, and Gao et al. [40] measured the average particle size of RP as 29.78 μm when the construction waste was produced by particle shaping. Zhao [4] used laboratory-discarded concrete beams, which were crushed by a jaw crusher, sieved with a 0.15 mm standard square-hole sieve, and finally ball-milled in a ball mill for 30 min, resulting in an RBP with an average particle size of 42.603 μm. Xue [41] crushed and sieved the RP obtained from the waste concrete pavement and measured the average particle size to be 30.2 μm. The experimental results of various scholars differed slightly, but the average particle size of the measured RP was concentrated in the range of 30–50 μm. The physical indicators of RP are summarized in Table 1 below.

### 3.2. Chemical Composition of RP

RP is mainly divided into RCP, RBP, and RMP. These three RPs have the same main chemical composition but different contents, and their main components are SiO_2_, CaO, and Al_2_O_3_, respectively [42,43,44]. The content of the three components exceeds 80% of the total composition. The content of SiO_2_ is the highest, accounting for about 50% of the total content, mainly from the aggregate of waste raw materials and cementitious material. They are followed by CaO, mainly from cementitious material. Finally, the content of Al_2_O_3_ ranks third because the bricks are not included in the cementitious materials. The specific composition of its chemical composition is shown in Table 2 below. In concrete applications, two components—SiO_2_ and Al_2_O_3_—are beneficial to the mechanical properties of concrete; SiO_2_ particles promote volcanic ash reactions, and Al_2_O_3_ contributes to the formation of calcium alumina and improves the early mechanical properties of the cementitious material [45]. Therefore, the early mechanical properties of concrete mixed with RBP are better than those of RCP and RMP under the same conditions.

### 3.3. Microscopic Analysis of RP

**Figure 6:**
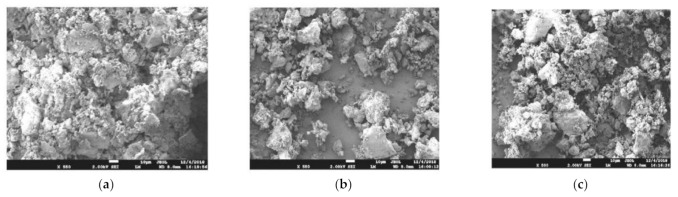
SEM images of different types of RP [54]. (**a**) RCP; (**b**) RBP; (**c**) RMP.

For the microscopic analysis of RP, Jin [49] used an X-ray diffractogram (X’Pert PRO MPD) to conduct phase analysis on the RP of construction waste. The results showed that the main phase of micro-powder of construction waste was SiO_2_, calcium silicate hydrate (C-S-H), CaCO_3_, and calcium aluminate hydrate (CAH). On the other hand, Yao [50], Zhang [51], et al. used an X-ray diffractogram to analyze the phase of the RP. The results showed that the main phase of the RP was SiO_2_ and CaCO_3_ and did not contain calcium silicate hydrate or calcium aluminate hydrate. The reasons for the above phenomenon are as follows: (1) the sources of RP are waste concrete and mortar, and its main components are SiO_2_ and CaCO_3_; (2) calcium silicate hydrate and calcium aluminate hydrate will react with CO_2_ in the air to produce SiO_2_ and CaCO_3_. Chen [52] observed the RP and the cement with an SEM electron microscope and found that compared to the cement particles, the RP particles are primarily irregular, with many fine particles attached to the surface, which is relatively rough and has a joint surface. Lv [53] used the Japanese JSM-7610F to analyze the micromorphology of RP and found that there were more sharp angles and irregular edges in the powder’s morphology. In addition, the surface was rough with a jagged microstructure, and Lv explained that this was the main reason for the reduced compatibility of the RP cement-based materials. Under the electron microscope, the particle morphology of RP powder is crumbly and angular, displaying irregular shapes. The surface is rough and lusterless, and the particle size varies. This microstructure causes RP’s workability and water retention to deteriorate, increasing water requirement. Figure 6 shows the microstructures of three different types of RP—RCP, RBP, and RMP—under SEM. The three samples were prepared by adjusting the ball mill speed and milling time to prepare three RPs with similar fineness [54]. The microstructures of all three RPs showed irregular shapes under SEM, with many tiny particles attached to the larger ones. The recycled brick powder had relatively good particle gradation, the angles were blunted, and the proportion of fine particles was moderate. This is because the clay minerals in the waste brick aggregate are soft and easy to grind. With the same fineness, the average particle size of the recycled brick powder is smaller, and the grading is better.

### 3.4. Activity Index

The activity index is a crucial indicator of gelling materials. According to the determination method of the activity index of RP in JG/T573-2020 “Recycled powder for Concrete and Mortar,” the RP is used instead of 30% cement to prepare mortar. After curing for 28 days, the compressive strength is measured after 28 days, and the 28 d compressive strength is compared to that of the mortar without RP to obtain the activity index of RP. Extension of the grinding time can increase the activity of the RP. The activity index of the initial RP was measured by Zhao et al. [55] as 55%, the activity index of the RP was 61% for 10 min of ball milling, and the activity index of the RP was about 72.6% for 30 min of ball milling. Kang [26] found that the activity index of RP increased from 55% to 71% after 30 min of grinding with a planetary ball mill. The particle gradation of RP affects the activity index of RP, and Fan [56] experimentally obtained that the activity index of RP was greater than 60% at 28 d. In contrast, the activity index of fly ash was relatively low. Y.H. [57] found that the activity index of 28d R.P. was higher than that of fly ash. The reasons for this situation are as follows: (1) The small particle size of RP can fill the gaps in cementitious material, which is conducive to developing the “micro-aggregates” filling effect. (2) The RP contains large amounts of Si, Al, Ca, and other elements necessary to form cementitious materials, so its activity index is extensive. The strength of the raw material used to prepare the RP also has an effect on the activity index of the RP, and Chen [58] measured that the 28 d activity indexes of the RP obtained from the preparation of C30 and C65 waste concrete were 75% and 80%, respectively. The type of grinding equipment has a significant impact on the activity index of the RP. Yu [19] used the airflow grinder to grind the RP, and the strength activity index of the RP was >0.7. According to [59], the RP activity index prepared by the ultrafine air grinder was higher than that of the RP prepared by vibration grinding.

According to the physical properties, chemical composition, microstructure, and activity index, the RP has a small particle size and a large specific surface area, which is conducive to the “micro-aggregates” filling effect. In addition to exhibiting periodic microstructure and high water absorption capacity, the activity index of RP is higher than that of fly ash. These factors make the RP potentially active, indicating the feasibility of RP as an auxiliary cementitious material. C.S. et al. [60] studied the correlation of RP and found that the average particle size of RP was small, the specific surface area was large, and the water absorption capacity was strong. They proposed the feasibility of RP as an auxiliary cementitious material.

## 4. Early Properties of RP Cement-Based Materials

### 4.1. Setting Time

**Figure 7:**
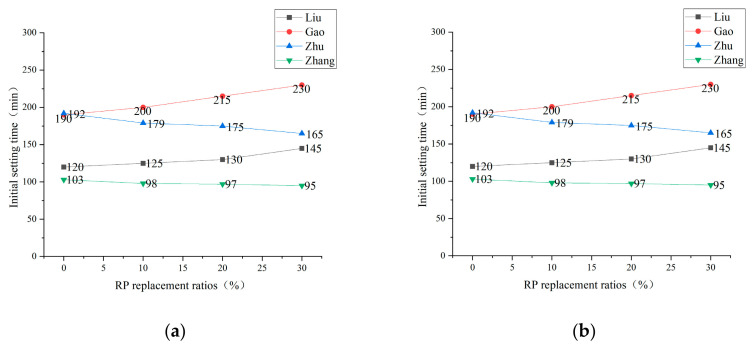
Setting time of net cement paste under different replacement ratios of RP [44,62,63,64]. (**a**) Initial setting time; (**b**) Final setting time.

With the hydration reaction, the net cement paste gradually loses its fluidity and plasticity and then solidifies into a hardened body with specific strength. The time used is the setting time. The setting time is one of the leading indicators to judge the early performance of cement-based materials. The addition of RP results in fewer cement particles for which hydration reactions can occur, producing less cementitious material and leading to longer setting times. For the longer setting time due to the admixture of RP, another view was presented by Zhang [61], who argued that the admixture of RP reduced the exothermic heat of the cement hydration reaction, which in turn delayed the process of early setting and hardening of the net cement paste. Liu [44] found that when 40% RP was added, the initial setting time and final setting time were delayed by 35 and 37 min, respectively, compared to the baseline group. Chen [35] found that with the increase in RP content, the initial setting time and final setting time of the net cement paste tended to be prolonged. On the contrary, Zhu [62], Zhang [63], et al.’s experimental study concluded that the addition of RP shortened the setting time of the net cement paste. Figure 7a,b summarizes the experimental data of several research scholars. It is found that the trend of setting time variation obtained by the researchers varies, but the extended or shortened time varies within a small range. The data all meet the requirements of setting the time of silicate cement in the specifications. When the amount of RP reaches 30%, the initial and final setting time of the net cement paste is extended or shortened to within 20%. On one hand, the cementitious property of RP is much less than that of cement. Incorporating RP will reduce the cement particles participating in the hydration reaction, reducing the amount of cementitious material generated. Moreover, it also negatively affects the intensity of the hydration reaction, reduces heat release, and delays the setting time of the net cement paste. On the other hand, the RP acts as a crystal nucleus and promotes the formation of hydration products. These two influencing factors cancel each other out, resulting in less influence of the addition of RP on the net cement paste.

### 4.2. Flowability

**Figure 8:**
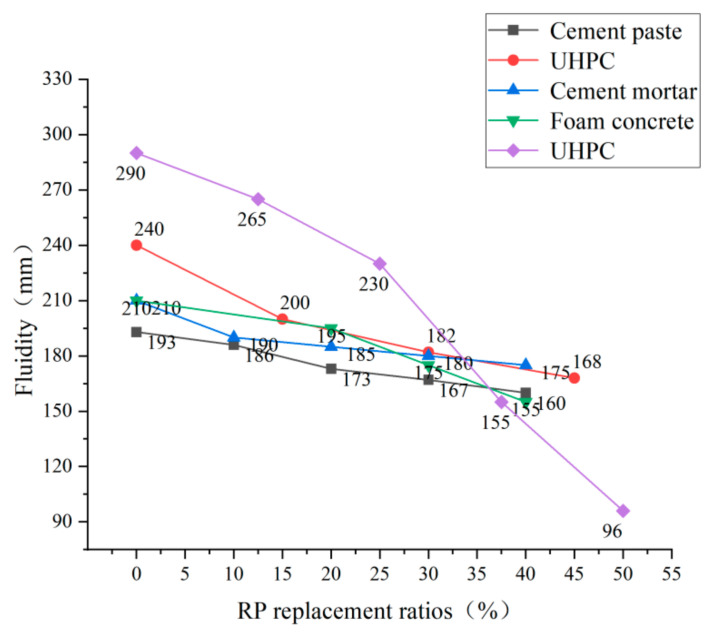
Fluidity of cement-based materials under different replacement rates of RP [44,52,67,68,69,70].

Fluidity is one of the indicators for evaluating the workability of cement-based material mixtures. Liu [44] found that the incorporation of RP reduced the flow of cement mortar. He attributed the reduction of cement mortar flowability to two reasons: (1) The RP particles are smaller than the cement particles and have a larger specific surface areas. Therefore, under the same water–cement ratio, the water content between the pores of the cement particles will be relatively reduced, resulting in a decrease in fluidity. (2) The small particle size of RP has a filling effect and fills between cement particles, which increases the resistance to the flow of net cement paste, thus leading to a decreased inflow. Xie [65] pointed out that the surface roughness of RCP and RBP increases the resistance of mortar particles to moving past each other. Guo [66] also came to the same conclusion in his study—that the RP harms the fluidity of mortar. The fluidity decreases with the increase in the RP content. Figure 8 summarizes the effects on the flowability of several cement-based materials after incorporating RP. It can be seen from the figure that the incorporation of RP adversely affects flowability. With the increase in the incorporated amount, the flowability of several cement-based materials shows a decreasing trend. For example, when the amount of RP is 30%, the fluidity of each cement-based material is reduced by between 10% and 25%. This is because the water absorption of RP is higher than that of cement. Therefore, the addition of RP leads to the reduction of free water content in cement-based materials. In addition, the RP particles are rougher and more irregular than cement particles from microstructural analysis, leading to more excellent frictional resistance to the relative sliding between particles. Therefore, the incorporation of RP harms the fluidity of cement-based materials.

### 4.3. Drying Shrinkage

**Figure 9:**
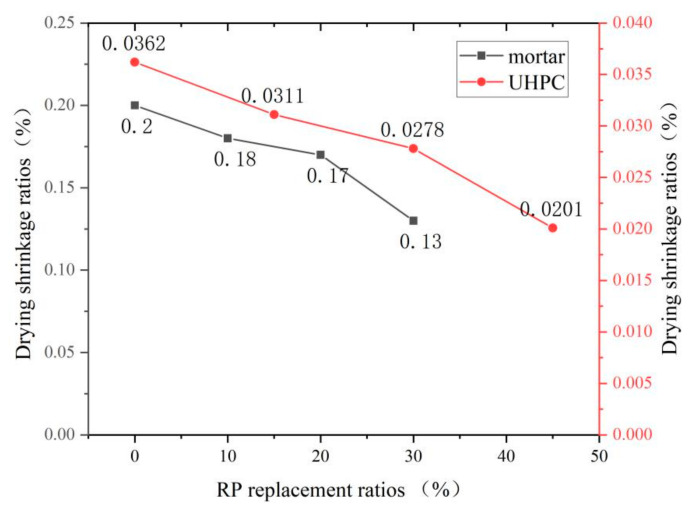
Drying shrinkage of mortar and ultra-high-performance concrete with different replacement rates of RP.

**Figure 10:**
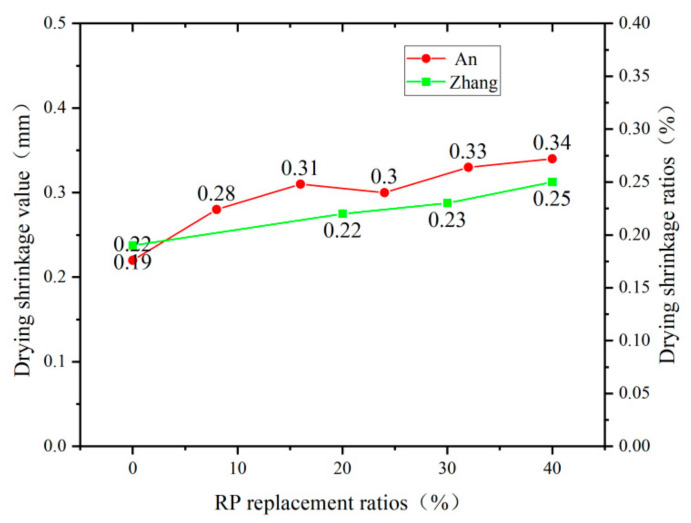
Drying shrinkage of foam concrete with different replacement rates of RP [43,68].

The effects of RP on the drying shrinkage of different types of cement-based materials are different. Gao [70] found that RP can effectively reduce the drying shrinkage of concrete by the circular method, and the finer the particles, the more pronounced the effect. Jiang [67] found a reduction in the drying shrinkage of ultra-high-performance concrete when increasing the RP replacement rate from 0 to 45%. However, the opposite conclusion was obtained when studying the drying shrinkage of RP foam concrete at different replacement rates. An [43] and Liu [71] found that the drying shrinkage value of RP foam concrete increases with the increase of RP admixture, and the trend of drying shrinkage growth gradually tends to be smooth with the extension of time. From Figure 9 and Figure 10, it can be seen that the addition of RP has opposite effects on the drying shrinkage of mortar, ultra-high-performance concrete, and foamed concrete. This phenomenon is because foam concrete has greater porosity and poor water retention compared to mortar and ultra-high-performance concrete. The heat generated by the hydration reaction of cement causes the water in the hydrated calcium silicate to evaporate, resulting in the migration of water in the specimen and generating a cascade of uncomplicated concave lunar surfaces. The capillaries then generate pressure to create water outflow. The admixture of RP further increases the porosity of the specimen, thus accelerating the water loss and therefore increasing the drying shrinkage of foam concrete [72]. The mechanism of adding RP to improve the drying shrinkage of mortar and ultra-high-performance concrete is: (1) The RP contains a large amount of inert material, which does not participate in the early hydration reaction after adding, reducing the content of hydration products, lowering the consumption of water, making its internal environment more temperate, and reducing drying shrinkage. (2) In the medium-term, the RP exerts a micro-aggregate filling effect, reducing the internal pore space of the material, improving its compactness, and enhancing its resistance to drying and shrinkage. (3) At a later stage, the RP releases the absorbed water, which further promotes the cement hydration reaction and produces more C-S-H gel, improving the internal pore space and increasing compactness [73].

## 5. Mechanical Properties of Cement-Based Materials with RP

### 5.1. Compressive Strength

Domestic and foreign scholars’ research on the compressive strength of RP cement-based materials mainly focuses on concrete, foam concrete, and ultra-high-performance concrete. The variables studied mainly focus on water–cement ratio, admixture amount, powder type, and fineness. Compressive strength is an important index reflecting the mechanical properties of cement-based materials, and the compressive strength under different research variables is not the same. The water–cement ratio plays a decisive role in the strength of concrete. Generally speaking, as the water–cement ratio of RP increases, its compressive strength tends to decrease [42]. However, it is not a simple linear relationship. In different water–cement ratio ranges, there are large differences in the impact of a water–cement ratio changes of 0.01. The degree of influence of recycled micronized powder admixture on the compressive strength of a high water–cement ratio is higher than on that of a low water–cement ratio. Cementitious materials with low water–cement ratios are more affected than those with a high water–cement ratio. This is because cementitious materials do not participate in the hydration reaction. These cementitious materials that do not participate in the hydration reaction will further react with the admixture of RP, which will weaken the adverse effect of the admixture of RP on the strength of the concrete [45].

**Figure 11:**
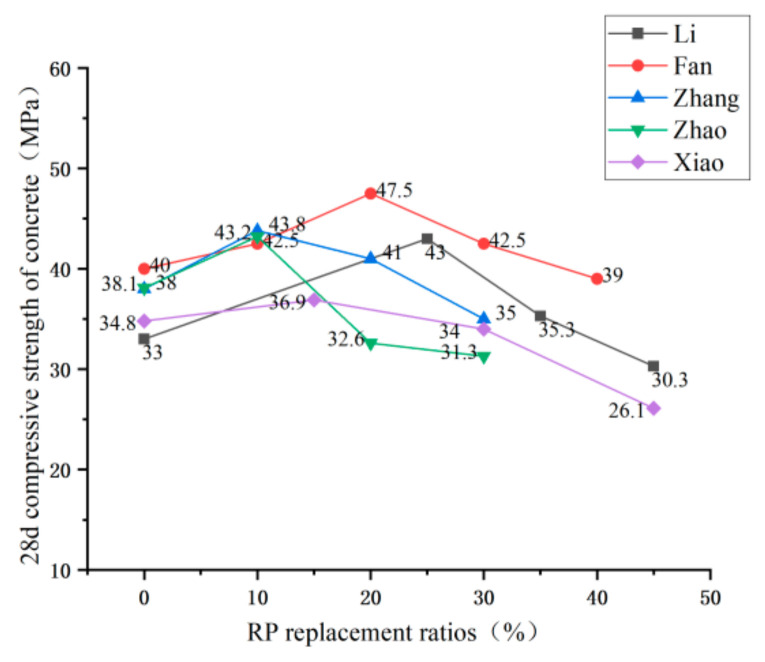
Compressive strength of concrete under different replacement rates of RP [27,42,74,75,81].

**Figure 12:**
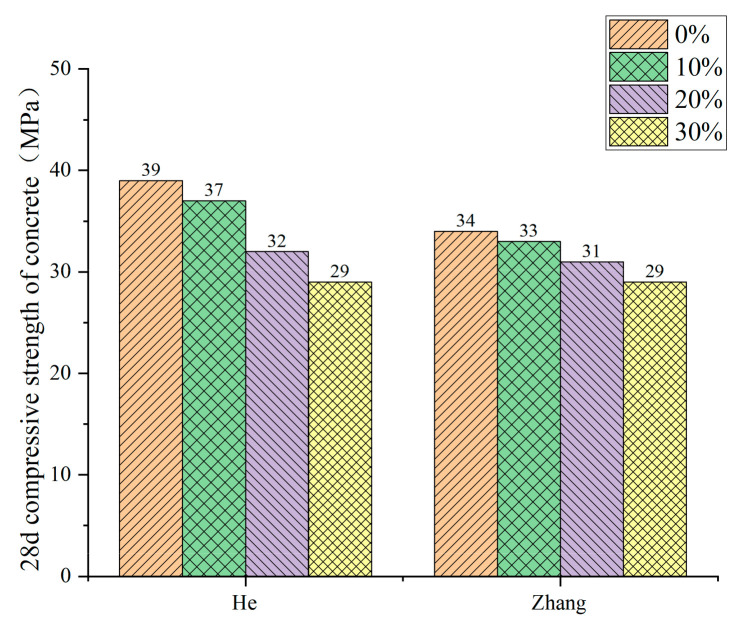
Compressive strength of concrete under different replacement ratios of RP [76,77].

**Figure 13:**
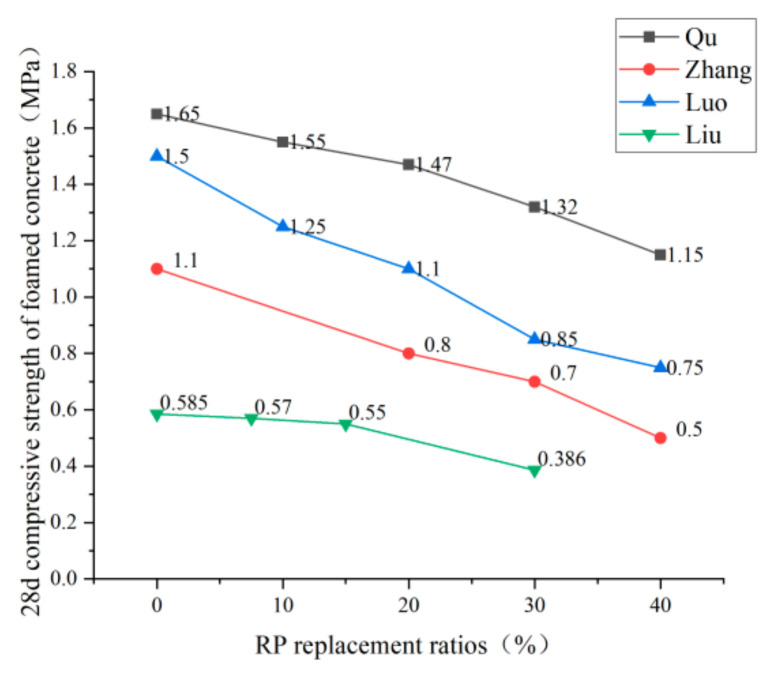
Compressive strength of foamed concrete under different replacement rates of RP [68,72,78,82].

**Figure 14:**
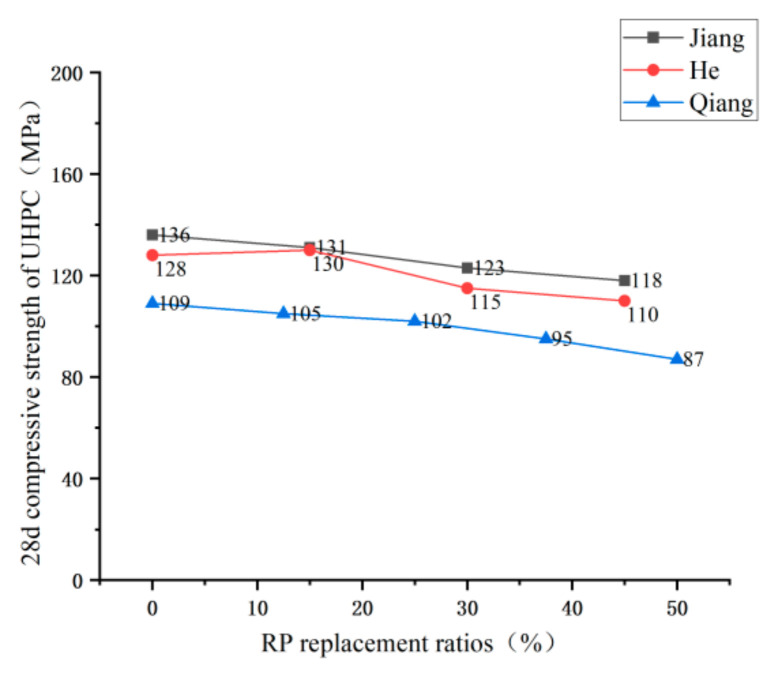
Compressive strength of UHPC under different replacement ratios of RP [67,69,79].

Domestic and foreign research scholars have not uniformly recognized the effect of RP admixture on the compressive strength of RP concrete; they can be broadly divided into having two conclusions. Li [42], Fan [74], and Zhang [75] et al., in their study, found that the RP could improve the compressive strength of concrete with a small dose but harm compressive strength with large dose. See Figure 11 for the trend. Under the conditions of a small dose, due to the small particle size of the RP, the volcanic ash reaction takes place to generate C-S-H gel, which fills the concrete pores and provides a specific compactness enhancement effect to the concrete. However, under the conditions of a large amount of RP, due to the defects of the RP itself—irregular particle shape, increased water demand under the same fluidity, and reduced cement content in concrete—the compressive strength of concrete is reduced. Other scholars came to different conclusions. He [76] and Zhang [77] et al. tested that the compressive strength of concrete presents a downward trend with the addition of RP. Under the conditions of small dose, the compressive strength of concrete can meet the requirements, reaching 80–90% of the compressive strength of ordinary concrete, and the trend is shown in Figure 12. They believe that the fewer active particles in the RP, which only play a micro-aggregate filling role in the net cement paste, positively affect concrete strength development. The under-hydrated cement minerals in the RP have a specific activity conducive to strength formation. Therefore, the strength change is not apparent under the conditions of a small dose. Figure 13 and Figure 14 summarize the influence of RP content on the compressive strength of foamed concrete and ultra-high-performance concrete, which is the same as the previous research results of Liu [71], Qu [72], Zhang [68], and Luo [78] et al. The compressive properties of the RP foamed concrete decrease slightly when the amount of RP is small, while the compressive strength of the RP foamed concrete decreases obviously when the amount of RP is large. For ultra-high-performance concrete (UHPC), the effect of RP admixture on UHPC is the same as the previous findings [67,79,80]. Therefore, considering the compressive strength of cement-based materials, the dosage of RP should not be too high. In the case of a small amount of RP, the positive effects of the volcanic ash reaction and crystal nucleation of the RP particles are apparent. The negative effect of the addition of RP manifests as a reduction in the amount of cementitious material; the two interact and restrict each other. Therefore, enhancing or weakening the compressive strength of cement-based materials is not apparent when the amount of RP is small. However, in the case of large dosage, the negative effect of incorporating RP to reduce the cementitious material played a leading role in the reaction, resulting in a significant decrease in compressive strength.

The type of RP affects the compressive strength of cement-based materials. The effect of RCP on the compressive strength of cement-based materials has been mentioned above. However, a consensus on the effect of RBP has not yet been reached among domestic and foreign scholars. Li [83] found that the 7 d compressive strength of concrete, with the increase in RBP replacement rate, first showed an increase and then a decrease, while the 28 d compressive strength showed a decreasing trend. The same finding was made by Che [84]—the incorporation of RBP reduces the compressive strength of concrete at 28 d. However, some scholars have found the opposite, and Sun [85] found that the 28 d compressive strength of self-compacting concrete with 1% to 5% RBP admixture was higher than the reference group. Lin [30] studied that the incorporation of RBP can improve the compressive strength of concrete. Hana [86] found that the compressive strength of mortar mixed with RBP was increased by 13%. The reasons that RBP enhances the mechanical properties of concrete are (1) the micro-aggregate filling effect of RBP improves concrete compactness. (2) The reaction of RBP with hydration product Ca(OH)_2_ produces C-S-H gel, which reduces the porosity of cement-based materials [87]. Du [48] studied the influence of the content of RBP on the compressive strength of UHPC and found that the compressive strength of UHPC decreased with the increase in content.

In addition, curing time impacts the compressive strength of concrete, and prolonging the curing time can alleviate the negative impact of RP on the compressive strength of cement-based materials. RP’s adverse effects on cement-based materials can be mitigated through methods such as improving the RP’s fineness and heat treating the RP.

### 5.2. Flexural Strength

**Figure 15:**
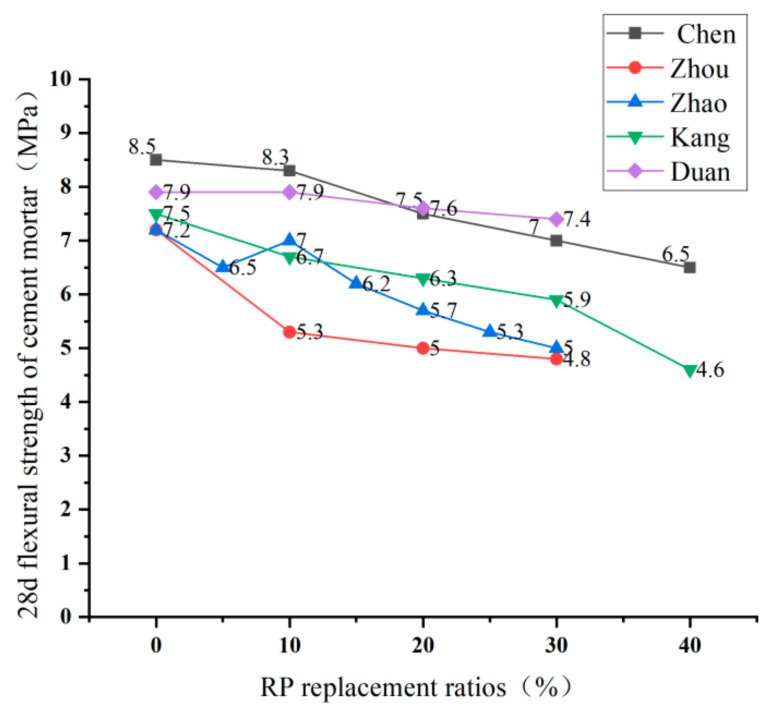
Flexural strength of cement mortar under different replacement ratios of RP [4,47,52,89,92].

The research on the flexural strength of cement-based materials by domestic and foreign scholars mainly focuses on cement mortar, and the research variables include the amount, ratio, and fineness of RP. Regarding the effect of RP content on the flexural strength of cement mortar, the research conclusions of domestic scholars are the same [46,47,88,89]. Figure 15 is a line graph based on the test data of domestic and foreign researchers. It can be seen from the figure that the flexural strength of cement mortar shows a downward trend with the increase in the content of RP, and the more the content is, the more pronounced the effect is. Because of the low activity of the RP, increasing the dosage harms the flexural strength. In terms of the effect of RP ratios on the flexural strength of cement mortar, Zhao [4] found that the effect of RP ratios on the flexural strength of cement mortar was not significant. Nevertheless, Liu [90] found that the 28 d flexural strength increased with increasing particle size when the majority of the RP was RCP and decreased with increasing particle size when the majority was RBP. For fineness, the influence of RP on flexural strength is similar to the effect on the compressive strength of cement-based materials. However, the effect of fineness on flexural strength is not as apparent as on compressive strength [91].

### 5.3. Splitting Tensile Strength

Splitting tensile strength is a measure of the tensile properties of concrete. The incorporation of RP adversely affects the splitting tensile strength of concrete, and the greater the amount of incorporation, the more obvious it is. Feng [38], Fang [93] and Xue [41] et al. found that the splitting tensile strength of recycled concrete showed an overall decreasing trend as the replacement rate of RP increased. However, an amount of admixture below 10% would not have a negative effect or could even have a slight benefit, and the strength decreased rapidly above 10%. An [43] investigated the effect of admixture amount on the splitting tensile strength of foam concrete and found that the substitution rate increased and the 7 d splitting tensile strength gradually decreased. The 28 d splitting tensile strength first increased slowly and then decreased rapidly. An appropriate proportion of compounding has a positive effect on the splitting tensile strength of concrete. For example, Liu et al. [94] found that when the substitution rate is 15%, the mass ratio m (RBP): m (RCP) was 6:4 with the incorporation of composite powder, which increased the splitting tensile strengths at 7 d and 28 d by 6.9% and 10.1%, respectively.

## 6. Linear Regression Analysis

In order to quantify the effect of the content of RP on the performance of cement-based materials, this paper further conducts data analysis based on previous experimental data. Among the many variables, a linear relationship was found between RP replacement ratios and the effects on four factors: the fluidity of cement mortar, the 28 d compressive strength of concrete and mortar, the 28 d compressive strength of foam concrete, and the 28 d flexural strength of cement mortar; the specific data are shown in Table 3 below. In order to further analyze the linear relationship between them, this paper relativizes the previous data to make the linear relationship more significant. The linear relationship between the content of RP and the relative fluidity of cement mortar, 28 d relative compressive strength of concrete and mortar, 28 d relative compressive strength of foamed concrete, and 28 d relative flexural strength of cement mortar will be introduced.

**Figure 16:**
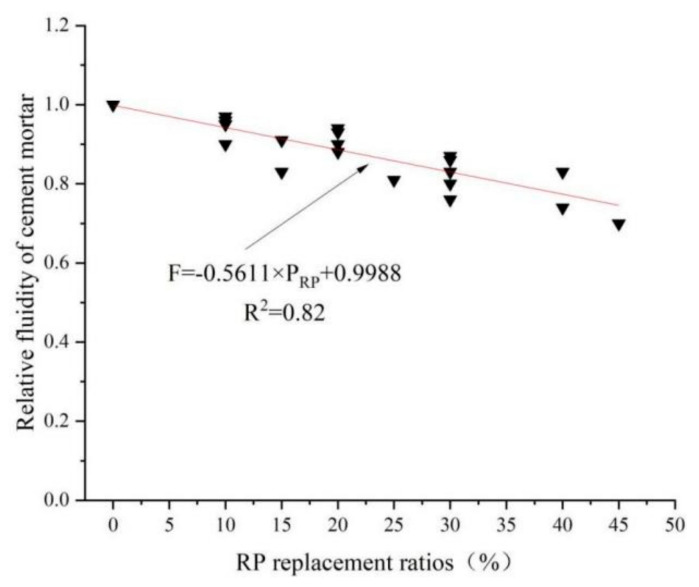
The relative fluidity of cement mortar under different replacement rates of RP [44,47,52,67,68,76].

Figure 16 shows the effect of the RP replacement rate on the relative fluidity of cement-based materials. The addition of RP reduces the fluidity of cement-based materials. There is a linear relationship between the content of RP and the relative fluidity of cement mortar, and the specific linear equation is as follows. In Equation (1), F represents the relative fluidity of cement mortar, F = F_x_/F_0_, F_x_ represents the fluidity of cement mortar under different RP replacement ratios, F_0_ represents the fluidity without RP, and P_RP_ represents the replacement ratios of RP.

F = −0.5611 × P_RP_ + 0.988 (R^2^ = 0.82) (1)

**Figure 17:**
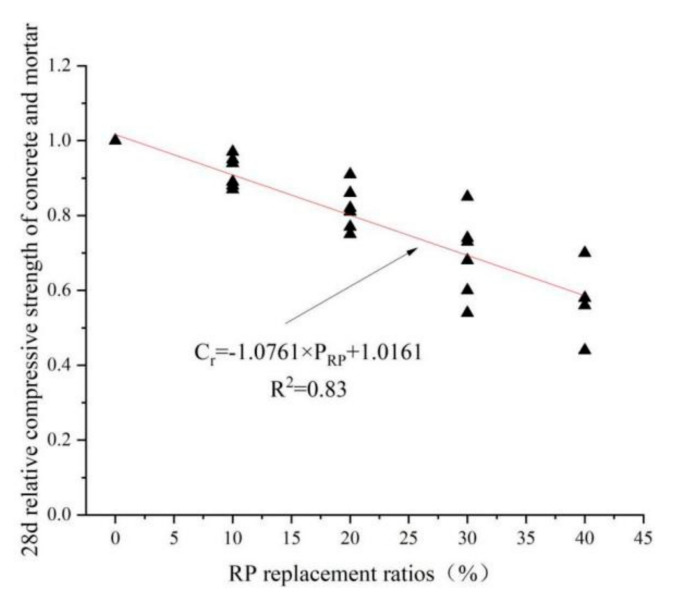
Graph of 28 d relative compressive strength of concrete and mortar under different replacement rates of RP [47,58,76,77,89,95].

**Figure 18:**
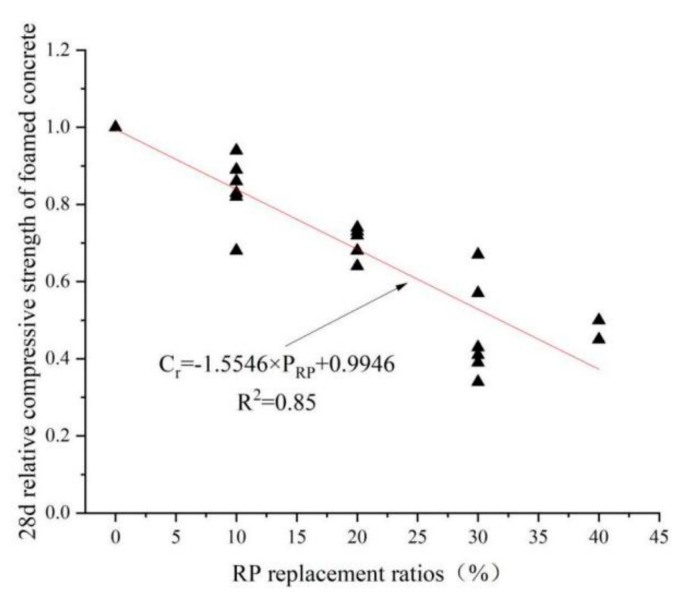
Graph of 28 d relative compressive strength of foamed concrete under different replacement rates of RP [46,68,72,78].

Figure 17 and Figure 18, using mathematical statistics and regression simulation, show a linear relationship between the RP replacement rate and the relative compressive strength of concrete and foam concrete, with the following linear equations. Equation (2) represents the relationship between the RP replacement rate and the 28 d relative compressive strength of concrete. Equation (3) represents the relationship between the RP replacement rate and foam concrete’s 28 d relative compressive strength. C_r_ represents the relative compressive strength, C_r_ = C_x_/C_0_, C_x_ represents the compressive strength at different RP replacement ratios, and C_0_ represents the compressive strength without RP. The slope of Equation (2) is significantly smaller than that of (3), which means that under the same premise, the adverse effect of the amount of RP on foamed concrete is more evident than on concrete.

C_r_ = −1.0761 × P_RP_ + 1.0161 (R^2^ = 0.83) (2)

C_r_ = −1.5546 × P_RP_ + 0.9946 (R^2^ = 0.85) (3)

**Figure 19:**
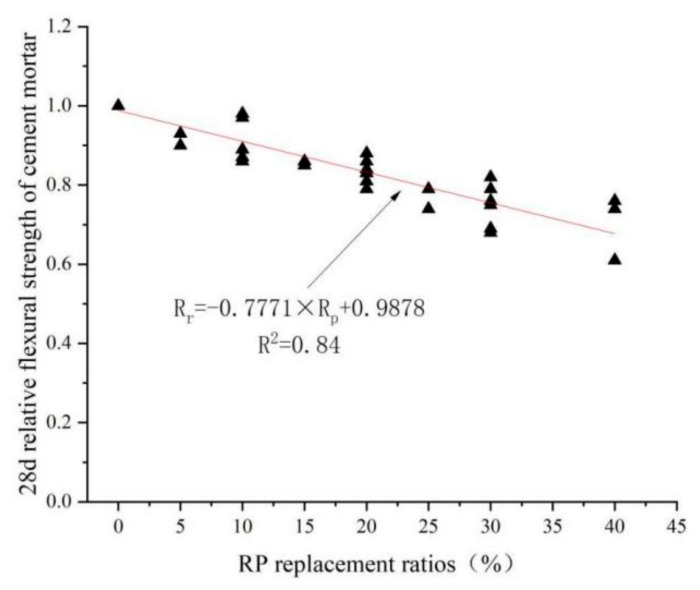
Graph of 28 d relative flexural strength of cement mortar under different replacement rates of RP [4,36,52,76,89,96,97].

Figure 19 further demonstrates the relationship between RP replacement rate and the relative flexural strength of cement mortar with the following linear equation. R_r_ represents the relative flexural strength, R_r_ = R_x_/R_0_, R_x_ represents the flexural strength under different replacement ratios of RP, R_0_ represents the flexural strength of cement mortar without RP, and P_RP_ represents the RP replacement ratios.

R_r_ = −0.7771 × P_RP_ + 0.9878 (R^2^ = 0.84) (4)

SPSS linear regression analysis was used to obtain the standard regression coefficients between RP and cement mortar content, as shown in Table 4. By comparing the absolute value of the standard regression coefficient, the influence degree of the mixing amount of RP on the four dependent variables can be judged. For example, it can be observed that the order of influence of the RP admixture is: 28 d relative compressive strength of foamed concrete > 28 d relative compressive strength of concrete and mortar > 28 d relative flexural strength of cement mortar > relative fluidity of cement mortar.

Considering that the difference between the standard regression coefficients simulated by SPSS linear regression is too small, the effect is insignificant. Therefore, this paper adopts the second method: the grey relational analysis method. Since there are noticeable differences in the values corresponding to each dosage of RP in the collected data, the optimal value is selected from the indexes corresponding to each dosage of RP for grey relational analysis, as shown in Table 5.

### 6.1. Matrix Dimensionless Processing

Since the data in Table 6 have been relativized (initialized) already, this step can be skipped.

$$(X)=\left[\begin{array}{ccccc} 1 & 0.97 & 0.91 & 0.87 & 0.83\\ 1 & 0.97 & 0.91 & 0.85 & 0.7\\ 1 & 0.94 & 0.74 & 0.67 & 0.5\\ 1 & 0.98 & 0.88 & 0.82 & 0.76 \end{array}\right]$$

Taking the amount of RP as the parent sequence, the relative fluidity of cement mortar, the 28 d relative compressive strength of foamed concrete, the 28 d relative compressive strength of concrete and mortar, and the 28 d relative flexural strength of cement mortar are the characteristic sequences.

### 6.2. Calculate the Relational Coefficient of Each Indicator

The absolute value of the difference between the reference sequences and the character sequences is calculated to determine the variance change matrix, the maximum value *δ*_max_, and the minimum value *δ*_min_ in the variance change matrix. The grey relational coefficient is determined according to $\xi =\frac{\delta_{\min}+\rho \delta_{\max}}{\delta +\rho \delta_{\max}}$, where ρ is the discriminant coefficient, taken as ρ = 0.50, and *δ* is each element in the difference matrix. The grey relational coefficient was calculated as shown in Table 6 below.

### 6.3. Calculate the Grey Relational Degree

The following equation calculated the grey correlation, and the relationship between the degree of influence was obtained by ranking the correlations in order of magnitude. The results are shown in Table 7.

$$\gamma_{i}=\frac{1}{m}\sum_{j=1}^{m}\xi (j)$$

where ξ(j) is the value of the grey correlation coefficient; m represents the number of values in each column.

The results of the gray correlation analysis and the results of the standard regression coefficients simulated by SPSS linear regression are the same, and the two methods corroborate each other. Therefore, the degree of influence of the amount of RP on the four factors is as follows: 28 d relative compressive strength of foamed concrete > 28 d relative compressive strength of concrete and mortar > 28 d relative flexural strength of cement mortar > relative fluidity of cement mortar. This relationship shows that under that exact dosage of RP, the adverse effect on the 28 d relative compressive strength of foamed concrete is the largest and the adverse effect on the relative fluidity of cement mortar is the least.

## 7. RP Activation Methods

The RP has a significant water demand, lower activity than class II fly ash, and poor adaptability to concrete admixtures. In addition, in contrast to cement, the strength of the cement-based material decreases with the increasing content of RP. These shortcomings limit the application of RP in practical engineering, so the activation of the activity of RP is a critical factor in improving the recycling efficiency of recycled construction waste. For this reason, many scholars in China and abroad are looking for ways to enhance the activity of RP. To summarize, here are five ways to activate RP:

(1) Increase the fineness of the RP. Increasing the fineness of the RP can increase the specific surface area of the RP, convert the stable α-SiO_2_ into amorphous SiO_2_, and then improve the activity of the RP [96,98]. Li et al. [99] believed that the particle size of the RP should be controlled to be less than 75 μm and that the finer the RP, the higher the activity. RCP powder with a particle size smaller than 75 μm promotes the formation of single-calcium carbonate. As the RP particle size decreases, the surface binding energy decreases, the Si-O and Al-O chemical-bond-breaking energy decreases, and the internal structure reorganizes, making it easier to generate cementitious substances. However, the RP should not be too fine, as the material will agglomerate, which will reduce the compactness of void filling and reduce the strength of the specimen [96,97,100].
(2) RP heat treatment. Florea [101] found that the 28 d compressive strength of RP mortar increased by 14.7% and 20.1% after high-temperature treatment at 500 °C and 800 °C, respectively. Kang [102] found that the excitation effect after treatment at 800 °C was better than that after treatment at 600 °C. He attributed this to the fact that the high temperature caused an activation effect of the RP similar to that of blast furnace slag. The RP produced a phase similar to that of calcium silicate in cement at a high temperature of 800 °C. Lv [103] measured the RP activity at 200 °C, 400 °C, 600 °C, and 800 °C and found that the RP activity first increased and then decreased with the increase in temperature, and the optimal thermal activation temperature was 600 °C. The cementitious material will coagulate and separate under high-temperature conditions. When the temperature is above 500 °C, CaCO_3_ will decompose into CaO. The decomposition of C-S-H and Ca(OH)_2_ in the cementitious material forms CaO, which has a high specific surface area and high activity. Generally speaking, the higher the CaO content in the RP, the greater the activity. However, scholars’ optimal heat treatment temperatures are inconsistent and are related to the source and composition of the RP used in the experiment.
(3) Alkali-activated treatment. The RP composition of calcium carbonate, calcium silicate, and silica was measured by EDS, providing a solid basis for alkali chemical excitation [58]. Dong [104] found that when 0% to 4.8% NaOH was added, the specimen strength increased with NaOH dosing and gradually decreased when it exceeded 4.8%. The addition of RP reduces the alkalinity of the concrete, and the addition of an alkali exciter can improve the activity of the RP. It can be explained that the addition of an alkali activator can promote the formation of C-S-H in concrete and make the microstructure of concrete denser. Liu [105] showed that the excitants Na_2_SO_4_, NaOH, Ca(OH)_2_, and NaHCO_3_ had specific active excitation effects on the RP through net cement paste specimen tests and scanning electron microscope observations. The optimal excitation doses were 3%, 3%, 2.5%, and 2.5%, respectively.
(4) CO_2_ treatment. Since the RP still has a large amount of calcium oxide, researchers have used CO_2_ to treat it RP. After carbonization, the strength of the cementitious material is higher than before carbonization. Maintenance with CO_2_ can improve the performance of the RP products. Li [106] treated the RP with a low concentration of CO_2_, which enhanced not only the physicochemical properties of the RP but also improved the activity of the RP. Cheng [107] found that the net cement paste mixed with carbonized cement paste powder had higher flow and compressive strength than that mixed with uncarbonized powder. This is because the addition of carbonized RP increases the calcium carbonate content in the system. The calcium carbonate reacts with the aluminum phase in the cement to form a hydrated calcium carbonate aluminate. This promotes the stability of ettringite and increases the volume of hydration products. The accelerated carbonation curing of concrete and mortar with CO_2_ allows them to gain strength quickly and have good mechanical properties and durability [108].
(5) Double mixing. RCP, RBP, fly ash, slag, and silica fume blending can improve the composition and promote the volcanic ash reaction of cementitious materials. It is conducive to improving the long-term strength of the concrete and can improve the workability of concrete. Xiao [109] showed that when RP was compounded with mineral powder, the early and late strengths were higher than those of single blending, and the late strength was more significantly improved later. This is because the RP and mineral powder produce a specific micro-aggregate effect. The mineral powder undergoes a secondary hydration reaction in the early and late stages under alkali substances, thus improving the concrete’s strength. Bai [110] studied the effect of compounding on the anti-carbonation performance of concrete. The compounding of RP, fly ash, and silica fume can significantly improve the anti-carbonation performance of concrete. The anti-carbonation performance of concrete is optimal when RP:(fly ash + silica fume) = 7:3. There are five ways to activate the RP.

## 8. Future Prospects of RP

(1) Regarding construction waste recycling, China has been vigorously promoting waste classification. However, the mechanism of waste concrete recycling is not perfect. At present, all waste is recycled together. The lack of inspection, other classification processes, and construction waste recycling negatively impact efficiency. China should establish construction waste recycling norms and further improve awareness of construction waste classification and recycling.
(2) At present, scholars in China and abroad mainly focus on studying the mechanical properties and workability of RP on cement-based materials but focus less on the durability of cement-based materials, especially corrosion resistance, and the need to strengthen the research on durability in the future.
(3) Concerning the optimum dose of RP for cement-based materials, the optimum dose obtained varies due to different initial conditions. For example, the type of RP, the fineness of the RP, the method of RP excitation, and the dosing of exciter will affect the optimum dose of RP for cement-based materials. Therefore, further research by researchers is required to make it a complete system.
(4) Regarding the activation method of RP, mechanical grinding, further research should be conducted to determine the optimal grinding time for different grinding machinery apparatuses and different types of RP. An industry standard must be developed and would be significant for reducing energy waste and for the industrial production of more efficient RP.
(5) Regarding the activation method of alkaline excitation treatment of RP, domestic and foreign scholars currently use single-activator activation, and research on composite-activator activation of RP is relatively scarce. Therefore, domestic and foreign scholars should increase research in this area.
(6) Scholars in China and abroad have concentrated on improving the mechanical strength of RP, and very few have studied the use of the low-strength properties of RP itself. For example, Xiao proposed to prepare low-strength foam recycled concrete from recycled raw materials and apply it to the material blocking system of the airport runway safety zone [111]. Scholars at home and abroad can also follow this idea to explore and study other uses of RP without deliberately pursuing high-strength and high-performance materials.
(7) Most researchers use the cementitious properties of RP to prepare cementitious materials. However, the cementitious properties of RP are far less than those of cement. Therefore, the performance of the prepared cementitious materials is not satisfactory. For example, it is feasible to combine RP with organic polymer material polyurethane, use quartz sand as coarse aggregate, RP as fine aggregate, polyurethane as cementitious material, and prepare a new material—RP polyurethane composite material.

## Figures and Tables

**Table 1:** Physical index of RP.

Average Particle Size (μm)	Apparent Density (kg/m^3^)	Specific Surface Area (m^2^/kg)	Bulk Density (kg/m^3^)
30~50	2300~2700	300~700	870~920

**Table 2:** Chemical composition of RP.

	SiO_2_	CaO	Al_2_O_3_	Fe_2_O_3_	MgO	K_2_O	Na_2_O	SO_3_	Source
RCP	50.93	18.18	13.55	6.37	2.75	3.50	2.45	0.92	[46]
RBP	60.56	10.07	17.16	3.46	1.78	2.50	1.26	0.34	[46]
RMP	56.64	17.41	10.54	5.59	1.24	3.53	3.02	0.92	[46]
RCP	62.18	10.24	10.15	2.49	1.91	1.73	1.30	0.47	
RBP	63.12	8.52	12.29	2.68	1.33	2.02	1.74	0.37	
RCP	48.25	27.55	11.09	4.94	2.42	2.25		1.20	[47]
RBP	67.83	1.67	16.20	7.55	0.94				[48]
RP	63.1	10.2	10.3	2.5	1.9	1.8	1.4	0.5	[32]
RP	47.9	18.7	12.0	6.53	2.26	2.33	0.86	1.41	[22]

**Table 3:** Summary of data related to RP.

	RP Replacement Ratios (%) —Data (Top–Down: mm; MPa; MPa; MPa)	Regression Equation (After Relativization)	Source
the fluidity of cement mortar	0–193; 10–186; 20–173; 30–167; 40–160	$\begin{array}{c} F=-0.5611\times P_{\mathrm{RP}}+0.9988\\ {\;R}^{2}=0.82 \end{array}$	[44]
	0–230; 10–219; 20–216; 30–184		[47]
	0–210; 10–190; 20–185; 30–180; 40–175		[52]
	0–240; 15–200; 30–182; 45–168		[67]
	0–210; 20–195; 30–175; 40–155		[68]
	0–210; 10–203; 15–191; 20–190; 25–187; 30–184		[76]
the 28 d compressive strength of concrete and mortar	0–39.2; 10–34; 20–31.7; 30–28.5	$\begin{array}{c} C_{r}=-1.0761\times P_{\mathrm{RP}}+1.0161\\ {\;R}^{2}=0.83 \end{array}$	[48]
	0–39; 10–37; 20–32; 30–29		[76]
	0–34; 10–33; 20–31; 30–29		[77]
	0–50.5; 10–47.5; 20–37.5; 30–27.5; 40–22		[89]
	0–65; 10–58; 20–50; 30–39; 40–38		[95]
	0–49.3; 10–43.6; 20–42.5; 30–33.6; 40–27.8		[58]
the 28 d compressive strength of foam concrete	0–1.12; 10–1.0; 20–0.83; 30–0.46 0–0.64; 10–0.55; 20–0.46; 30–0.43 0–1.11; 10–0.76; 20–0.71; 30–0.48 0–1.09; 10–0.89; 20–0.74; 30–0.42	$\begin{array}{c} C_{r}=-1.5546\times P_{\mathrm{RP}}+0.9946\\ {\;R}^{2}=0.85 \end{array}$	[46]
	0–1.1; 20–0.8; 30–0.7; 40–0.5		[76]
	0–1.65; 10–1.55; 20–1.47; 30–1.32; 40–1.15		[72]
	0–1.5; 10–1.25; 20–1.1; 30–0.85; 40–0.75		[78]
the 28 d flexural strength of cement mortar	0–7.2; 5–6.5; 10–7; 15–6.2; 20–5.7; 25–5.3; 30–5	$\begin{array}{c} R_{r}=-0.7771\times R_{P}+0.9878\\ {\;R}^{2}=0.84 \end{array}$	[4]
	0–7.8; 10–6.7; 20–6.3; 30–5.3		[36]
	0–8.5; 10–8.3; 20–7.5; 30–7; 40–6.5		[52]
	0–6.2; 10–5.4; 15–5.3; 20–5.0; 25–4.9; 30–4.7		[76]
	0–7.5; 10–6.7; 20–6.3; 30–5.9; 40–4.6		[89]
	0–8.8; 10–8.3; 20–7.5; 30–6.7; 40–6.5		[96]
	0–3.75; 5–3.5; 10–3.28; 20–3.11		[97]

**Table 4:** Standardized regression coefficients.

Independent Variable	Dependent Variable	Standardized Regression Coefficients	Significance
RP content	relative fluidity of cement mortar	−0.907	1.8107 × 10^−10^
	28 d relative compressive strength of concrete and mortar	−0.914	1.8161 × 10^−12^
	28 d relative compressive strength of foamed concrete	−0.924	1.6625 × 10^−11^
	28 d relative flexural strength of cement mortar	−0.917	3.7291 × 10^−15^

**Table 5:** Optimal values under each index.

RP Content (%)	Relative Fluidity of Cement Mortar	28 d Relative Compressive Strength of Concrete and Mortar	28 d Relative Compressive Strength of Foamed Concrete	28 d Relative Flexural Strength of Cement Mortar
0	1	1	1	1
10	0.97	0.97	0.94	0.98
20	0.91	0.91	0.74	0.88
30	0.87	0.85	0.67	0.82
40	0.83	0.7	0.5	0.76

**Table 6:** Grey relational coefficient.

Relative Fluidity of Cement Mortar	28 d Relative Compressive Strength of Concrete and Mortar	28 d Relative Compressive Strength of Foamed Concrete	28 d Relative Flexural Strength of Cement Mortar
0.40	0.40	0.40	0.40
0.44	0.44	0.45	0.44
0.50	0.50	0.58	0.51
0.56	0.57	0.69	0.59
0.65	0.75	1	0.70

**Table 7:** Grey relational degree.

Evaluation Items	28 d Relative Compressive Strength of Foamed Concrete	28 d Relative Compressive Strength of Concrete and Mortar	28 d Relative Flexural Strength of Cement Mortar	Relative Fluidity of Cement Mortar
Grey relational degree	0.623	0.531	0.526	0.508
Ranking	1	2	3	4

## Data Availability

All data generated or analyzed during this study are included in this published article.
